# Clinical effect of catgut implantation at acupoints for the treatment of simple obesity: A multicentre randomized controlled trial

**DOI:** 10.1097/MD.0000000000023390

**Published:** 2020-11-25

**Authors:** Xia Chen, Wei Huang, Dan Wei, De-Guang Ding, Yang Jiao, Hong-Ling Pan, Yi-Ting Jin, Yi-Wei Zheng, Yan-Ji Zhang, Ying-Rong Zhang, Yi-Ran Liu, Zhong-Yu Zhou

**Affiliations:** a Department of Acupuncture, Hubei Provincial Hospital of Traditional Chinese Medicine; b Hubei Province Academy of Traditional Chinese Medicine; c Hubei University of Chinese Medicine/The Co-innovation Center for Preventive Treatment of Disease of Acupuncture-moxibustion in Hubei Province, Wuhan, China.

**Keywords:** acupuncture, catgut implantation at acupoints, clinical study, obesity

## Abstract

**Background:**

Catgut implantation at acupoints (CIA) is a subtype of acupuncture that has been widely used to treat simple obesity, but evidence for its effectiveness remains scarce. The aim of this study is to evaluate the efficacy and safety of treating simple obesity with CIA.

**Objective:**

This clinical trial aims to evaluate the effectiveness and safety of CIA used for treatment of simple obesity.

**Methods:**

This is a multicentre, randomized, parallel, sham-controlled clinical trial. A total of 216 patients with simple obesity will be recruited. They will be randomly assigned in a 1:1 ratio to either the CIA group or the sham control group. All treatments will be given once every 2 weeks. The primary outcome measure is the rate of waistline reduction. Secondary outcome measures are the rates of reduction of body measurements, including weight, body mass index (BMI), hipline, waist-hip-ratio (WHR) and body fat percentage (BFP), the changes in scores on scales, including the Impact of Weight on Quality of Life Questionnaire (IWQOL-Lite), Short Form 36 (SF-36), the Hospital Anxiety and Depression Scale (HAD) and the Self-Esteem Scale (SES), Outcomes will be evaluated at baseline and at weeks 4, 8, 12, 16, 28, and 40, respectively. All adverse events that occur during this study will be recorded. If any participant withdraws from the trial, an intention-to-treat analysis (ITT) will be performed.

**Conclusion:**

This is a randomized, sham-controlled trial of CIA treatment for simple obesity. The results of this trial will provide more evidence on whether CIA is efficacious and safe for treating obesity.

**Trial registration:**

ClinicalTrials.gov Identifier: NCT02936973. Registered on October 18, 2016.

## 1 Introduction

Obesity is a type of chronic metabolic disease characterized by the excessive accumulation or abnormal distribution of fat in the body. Simple obesity is a type of obesity caused simply by energy intake exceeding energy consumption, in which other diseases or medical factors are excluded.^[1]^ With the development of the social economy and changes in lifestyle, the morbidity of obesity is increasing annually. According to the World Health Organization, the global overweight and obese populations, are predicted to reach more than 2.16 billion and 1.12 billion, respectively, by 2030.^[2]^ In 2008, the overweight and obese populations in China rose to 25.4% and 5.7%, respectively. In 2020, the obese population of China may be larger than that of America.^[3]^ Many studies^[4,5]^ have demonstrated that obesity is not only closely related to diabetes, polycystic ovary syndrome and other metabolic diseases but also an independent risk factor for hypertension, hyperlipidaemia, atherosclerosis and other metabolic diseases.

Current treatments for obesity mainly include drug treatments, operative treatments, changes in lifestyle (exercise, diet control), and traditional Chinese medicine (TCM). The use of pharmacological drugs for managing obesity remains controversial due to the side effects of these drugs.^[6,7]^ Gastrointestinal surgery is associated with high costs, risk of complications and strict indications. Acupuncture, which originated in China and is based on the framework of TCM, is an important alternative and complementary therapy for treating numerous diseases. As an available and affordable treatment, acupuncture is popular with many obese patients and has been widely used worldwide.^[8–10]^

As a subtype of acupuncture, CIA has developed rapidly over the last 60 years, and has been shown to have specific effects on chronic diseases, including simple obesity. Compared with manual acupuncture, the advantages of CIA lie in its persistence, improved patient compliance and superior long-term effects.^[11]^ A meta-analysis of CIA for obesity revealed that CIA is equivalent to drugs based on an assessment of pooled outcomes (improvement rate, weight loss, BMI, waistline, and hipline).^[12]^ Other recent meta-analyses^[13,14]^ indicate that treating obesity with CIA has a higher clinical effective rate than manual acupuncture and a rate equal to that of electro-acupuncture. Additionally, this treatment for treating obesity has positive effects on menopathy, hyperglycaemia, hyperlipidaemia, hypercholesterolemia and gastrointestinal disease, which has fewer adverse effects and more cost-effective.^[15]^ Treating obesity with CIA is likely to be applied more widely in the future. Although CIA has been used to treat obesity for several years, clinical trials have small sample sizes or other methodological limitations. Thus, a high level of evidence for CIA in the treatment of obesity must be obtained.

Most clinical trials investigating the treatment of simple obesity with CIA have compared CIA with manual acupuncture and electro-acupuncture,^[14]^ which led to 3 key questions: Is CIA absolutely effective and safe? Is the efficacy of CIA due to the effect of needling or blood-pricking and does this treatment for obesity result in a placebo effect? Does CIA have other effects (such as improving insulin resistance due to simple obesity and the quality life of obese people) in addition to helping obesity patients reduce their waistline measurements and lose weight?

The international gold standard remains to evaluate intervening measures with scientifically and normatively designed randomized, double-blind, sham-controlled trials.^[16]^ Due to the nature of CIA, it is not feasible to blind the acupuncturist during this study. However, all patients, statisticians and evaluators will be blind. To answer the above 3 questions, we wish to design a randomized, parallel, multicentre trial at 3 sites in China with an appropriate sample size using sham control to demonstrate its safety and efficacy.

## 2 Methods/design

### 2.1 Ethics approval

The study design complies with the guidelines of the Declaration of Helsinki (Version Edinburgh 2000), meets medical ethics requirements and has been approved by the Ethics Committee of Hubei Provincial Hospital of Traditional Chinese Medicine (HBZY2016-C20–01), Dongzhimen Hospital Beijing University of Chinese Medicine (DZMEC-KY-2017–15) and the First Hospital of Hunan University of Chinese Medicine (HN-LL-KY-2017–001–01). The study has been registered under the identifier NCT02936973 at Clinical Trials.gov.

### 2.2 Study/design

**Figure 1:**
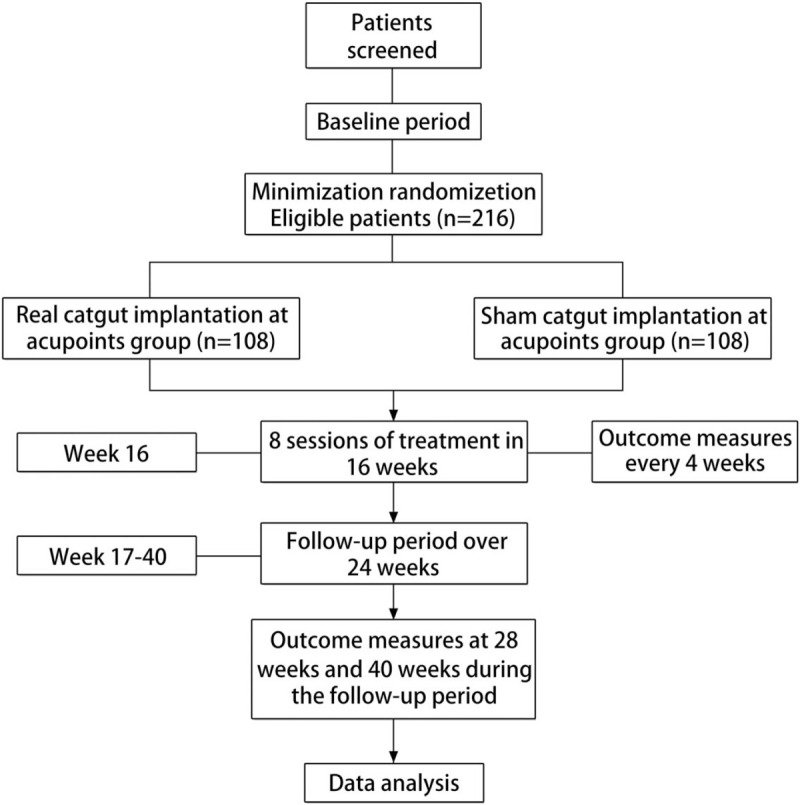
Flowchart for the trial.

This randomized, controlled, two-arm clinical trial will be conducted at Hubei Provincial Hospital of TCM, Dongzhimen Hospital Beijing University of Chinese Medicine and the First Hospital of Hunan University of Chinese Medicine. This study includes the following scheduled events: a treatment period of 16 weeks and a follow-up period of 24 weeks. The total study period will be 40 weeks. During the baseline period, each participant should keep a diet and exercise diary for 2 weeks before randomization. The diary will be kept throughout the research cycle if the participant is eligible for the study. The patients will be randomly selected for the CIA group (n = 108, expected) or the sham control group (n = 108, expected). CIA will be administered once every 2 weeks. All patients will receive 16 weeks of treatment. Each patient's blood pressure, heart rate, routine blood parameters and hepatorenal function will be tested before randomization and at the end of treatment. Body measurements of obese people (waistline, weight, BMI, et al) should be conducted and the IWQOL-Lite,^[17]^ SF-36,^[17]^ HAD,^[18,19]^ and SES^[20]^ will be completed throughout the study. Figure 1 provides a flow chart for the study.

### 2.3 Participants

#### 2.3.1 Diagnostic criteria

Patients must meet the diagnostic criteria in the guideline of The Asia-Pacific perspective: redefining obesity and its treatment.^[21]^ According to the guideline, the standard for Asian adult obesity is BMI≥25, whereas, for European adults, obesity is defined by BMI≥30. The difference is due to racial and epidemiologic diversity. Owing to this study will be conducted in China, the Asian obesity standard is chosen. Because early treatment is superior when TCM is used, 1 inclusion criterion for this study is a BMI between 25 and 30.

#### 2.3.2 Inclusion criteria

1. Diagnosed with simple obesity^[21]^
2. Age: 18–45
3. BMI: 25≤BMI<30
4. Waistline: males≥95 cm; females≥85 cm
5. Smokers have not changed their smoking habits for at least 2 months^[22]^
6. Willing to accept the above-described intervention methods

#### 2.3.3 Exclusion criteria

1. Endocrine disease (such as polycystic ovary syndrome, Cushing's disease, or hypothyroidism)
2. Gestational diabetes or uncontrolled hypertension (SBP≥160 mm Hg; DBP≥100 mm Hg),^[23]^ or lung, heart, liver or kidney disease
3. Nervous system disease or mental disorders, history of hospitalized depression, 2 instances of paralepsy or suicidal tendencies^[24]^
4. History of clinical diagnosis of an eating disorder,^[24]^ such as bulimia,^[25]^ cynorexia or anorexia; weight changes greater than 5 kg in the previous 3 months
5. History of weight loss with surgery^[26]^ or a history of post-operative adhesion^[25]^
6. History of taking taken drugs with a known influence on weight or appetite in the previous 3 months, such as diet pills, corticosteroids, anti-depressants, diazepam, nonselective body antihistamines, nicotine replacements, or hypoglycaemic drugs or planning to give up smoking and drinking
7. Pregnancy, lactation, or planning to become pregnant within 40 weeks
8. Received CIA
9. Participated in clinical research on obesity in the previous 3 months^[27]^
10. Protein allergies and scars
11. Skin disease such as eczema and psoriasis
12. Coagulation disorders, or use of warfarin, heparin or other anticoagulant drugs
13. Not able to cooperate or maintain the treatment during the study period

#### 2.3.4 Withdrawal from the study

1. A specialist physician at each centre responsible for evaluating serious adverse effects during the study decides to withdraw the patient from the study
2. Severe complications or other severe diseases necessitating emergency measures
3. Participants decision to withdraw from the study

#### 2.3.5 Recruitment

We will design a public recruitment advertisement and recruit any patients who meet the criteria through newspapers, posters, WeChat or the Internet. The doctor will determine whether the patients are eligible to participate in this study. All patients will be given sufficient time to decide whether to participate in the trial. A voluntarily signed, written, the informed consent form will be obtained from each participant. It provides the patients with details about related clinical research in language that the patients or their legal representatives can understand, including descriptions of the research objective, the research method and the process (including treatment measures, grouping, tests, potential benefits to the patients and potential risks and inconvenience that the patients may endure).

#### 2.3.6 Safety assessment

The safety assessment of CIA will include 3 aspects. First, a general physical examination that includes blood pressure, heart rate, routine blood, and hepatorenal function will be used as a safety index and be conducted before randomization and at the end of treatment. Besides, the incidence of adverse events, such as severe pain, allergic reaction, syncope, local induration, local haematoma, and local infection, will be recorded. The Visual Analogue Scale (VAS) will be used to assess the perception of pain due to CIA in the study.

### 2.4 Intervention

This is a placebo-controlled, double-blind, randomized trial, with a treatment of 16 weeks and a follow-up of 24 weeks. Each session will last for close to 10 minutes once every 2 weeks. All participants will be treated continuously for 16 weeks. The design of the study is consistent with the Standards for Reporting Interventions in Controlled Trials of Acupuncture (STRICTA) guidelines^[28]^ and the guidelines for clinical research on acupuncture (WHO Regional Publication, Western Pacific Series No.15, 1995). We will formulate standard operating procedures (SOPs) to ensure the standards and quality of the study. CIA will be performed by qualified TCM doctors with at least 3 years of clinical acupuncture experience.

#### 2.4.1 CIA group

Treatment measures for the CIA group will include CIA and lifestyle modification. The CIA prescription was selected by 3 steps: first, the domestic and overseas literature from over the last 30 years on CIA for curing simple obesity was reviewed. Based on complex network technology, the rules for selecting acupoints for treating simple obesity with CIA were analyzed. Subsequently, the aetiology and pathogenesis of obesity will be discussed by professional acupuncturists to modify the prescription according to basic theories of TCM. After the CIA, the stimulation will last for about 2 weeks. Two groups of acupoints will ultimately be selected for alternative treatment once every 2 weeks. The acupoints of Groups A and B are one to one in the same regions of the body. Group A includes Zhigou (TE6), Tianshu (ST25), Weishu (BL21), Zhongwan (CV12), and Zusanli (ST36); Group B includes Quchi (LI11), Huaroumen (ST24), Pishu (BL20), Shuifen (CV9), and Fenglong (ST40). There will be 8 treatments throughout the study.

##### 2.4.1.1 Application of catgut embedding

The instruments that will be used in the study include disposable embedding needles (#9#, Zhenjiang Gaoguan Medical Appliance Factory, Jiangsu, China), absorbable catgut sutures (000, Shandong Boda Medical Supplies Co. Ltd., Shandong, China), and disposable embedding aids (Yangzhou City Dragon Tiger Medical Instrument Factory, Jiangsu, China). CIA will be performed for patients assigned to the CIA group according to the Operation Standard for Acupuncture, Part 10: Catgut Implantation at Acupoints (GB/T 21709.10–2008). The doctors should wash both hands with soap, flush with running water and wipe with 75% ethyl alcohol and 0.5% iodophor before putting on sterile gloves. The operation site should be disinfected with 0.5% iodophor in a circular pattern from the centre outward. When the acupoints and the surrounding skin have been disinfected, an absorbable catgut suture of an appropriate length will be placed at the front end of the trocar before the stylet is connected. The proposed acupoint will be fixed with the thumb and index finger of one hand, and the needle will be inserted into the acupoint with the other hand to the required depth. Appropriate twirling, lifting and thrusting will be applied. When a needling sensation occurs, the stylet will be pushed while the tube is withdrawn, which will embed the absorbable catgut suture into the muscular layer or subcutaneous tissue of the acupoint. Then, the acupoint will be pressed with a dry cotton ball to avoid bleeding after the needle is withdrawn. The patient will be told to keep the skin at the acupoint dry for 3 days after treatment.

##### 2.4.1.2 Lifestyle modification

The lifestyle modification treatment will be designed by experienced nutritionists. First, the basal metabolic rate (BMR) of the patient will be calculated using the formula suggested by the FAQ/WHO. Second, the difference between the daily dietary caloric intake and calories consumed during exercise will be calculated according to the BMR, namely dietary caloric intake - calories consumed during exercise ≤ basal metabolic rate. Finally, a proper diet and exercise treatment will be designed for the patient based on the above difference. The dietary distribution will be designed according to the Chinese Dietary Guidelines approved and issued by the Standing Council of the Chinese Nutrition Society. The 3 nutrients included in the total energy should be in the following proportion: carbohydrates account for 55% to 65%, oil or fat accounts for 20% to 30%, and protein accounts for 15%. These will be distributed proportionally among 3 meals (breakfast 30%, lunch 40%, and dinner 30%) to distribute the energy throughout the day. In terms of daily exercise, brisk walking, jogging and swimming could be performed under the guidance of the physician. During the treatment, a diet and exercise diary will be distributed to the patients,^[29]^ and detailed guidance concerning their diet and exercise will be provided using an instruction manual for dining and sports. The patients should be encouraged to track their diet and exercise at least 4 days per week (3 days on weekdays and 1 day on the weekend).^[30]^ The meals and excise will be quantified using calories in the diary. The diet and exercise diary will be collected from each patient at baseline and at weeks 4, 8, 12, 16, 28, and 40 to estimate the intake of nutrients and to monitor the diet and amount of exercise. During the treatment, the patients will receive 16 consultations with a nutritionist. In addition, social tools (QQ and WeChat) will be used to build a social network among the doctors and patients during the 16 weeks of treatment and the 24 weeks of follow-up.

#### 2.4.2 Sham control group

**Figure 2:**
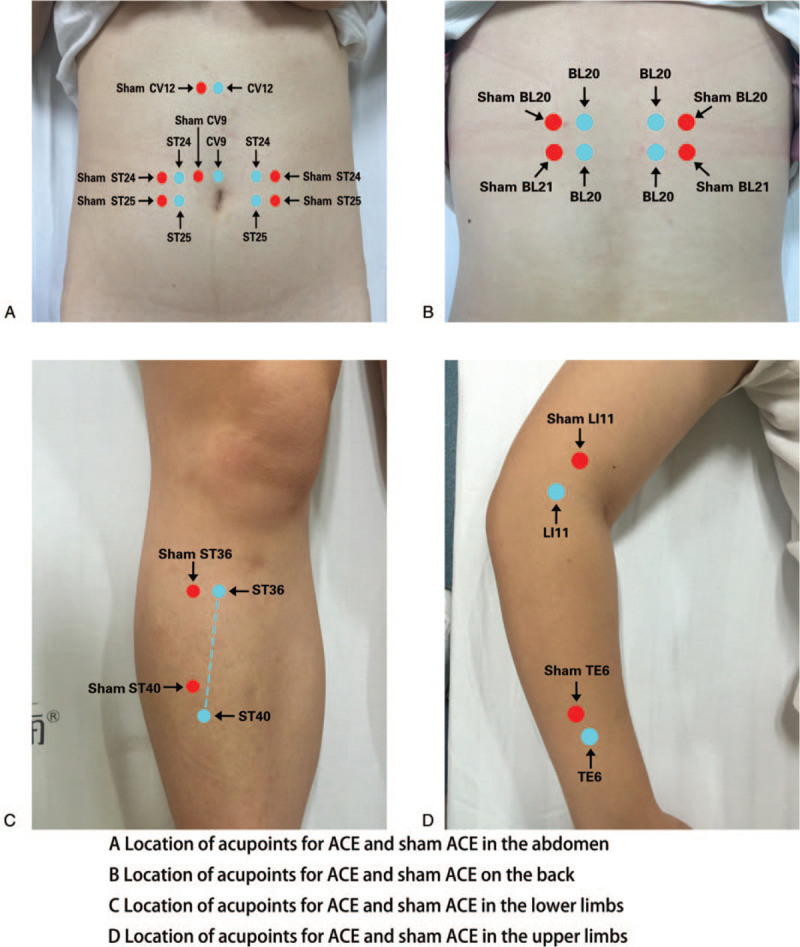
Acupoints for catgut implantation and places for control stimulation.

**Figure 3:**
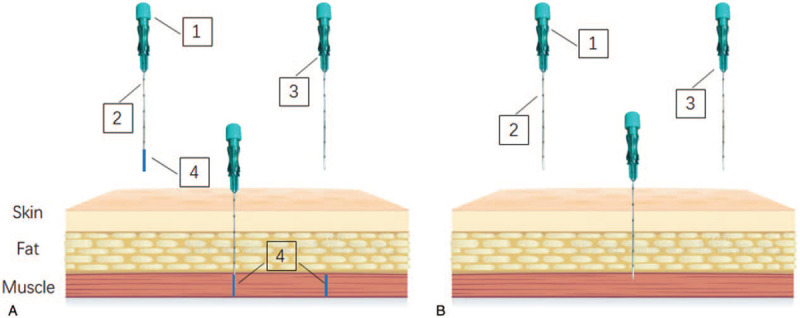
The difference between CIA group and sham CIA group.

The treatment in the sham control group will include sham stimulation and lifestyle modification. Acupoints for the sham control group are located 1 cun (≈20 mm) from the points used for the CIA group, and the position of sham acupoints cannot be located in any real acupoints and meridians of the body. The operation process will be similar to the one used with the CIA group. The patients will lie on their backs to expose the implantation locations. The positions will be located and disinfected with iodine. An empty needle will be pushed into the chosen position, to a depth equivalent to that used for CIA group. To ensure blinding, all patients will undergo the scheduled treatment. The physicians cannot communicate with the patients during the operation. The lifestyle modifications of the sham control group will be equivalent to those of the CIA group. The locations of CIA group and the sham control group are detailed in Figure 2. Figure 3 shows the difference between CIA group and sham CIA group. (A) A thread- embedding needle will be inserted in the location of acupoints. The catgut suture will be pushed into the tissue using the stylet. (B) A thread- embedding needle will be inserted into sham point, and catgut will be not embedded. (1.stylet 2.trocar 3.thread- embedding needle 4. surgical suture).

### 2.5 Outcome measurements

#### 2.5.1 Primary outcome

The primary outcome measurement of this study will be the rate of waistline reduction compared with the baseline. Evaluations will be conducted at weeks 0, 4, 8, 12, 16, 28, and 40. The waistline is the simplest and most practical index to measure the amount of fat accumulated in the stomach (central obesity). The distribution of fat in the body, especially the accumulation of fat in the stomach, has a close relationship with obesity-related diseases.

#### 2.5.2 Secondary outcome measurements

1. The reduction rate of weight, BMI, hipline, WHR, and BFP will be evaluated at baseline, and at weeks 4, 8, 12, 16, 28, and 40. These indicators are related to the shapes of obese people. High indicators suggest an increased risk of obesity-related diseases.
2. Changes in the SF-36, HAD and SES scores will be evaluated at baseline, and at weeks 16 and 40, and the IWQOL-Lite will be evaluated at baseline and at weeks 4, 8, 12, 16, 28, and 40. The SF-36 and IWQOL-Lite will be used to evaluate changes in the quality of life of the obese patients. The HAD and SES will be used to evaluate changes in the psychological status of the 216 obese patients.

### 2.6 Sample size estimation

Based on a review of the literature, clinical experience and discussion among clinical experts, we anticipated 7% waistline reduction at 16 weeks in the CIA group and 2% reduction in the sham CIA group. Eighty six participants per group was required based on:

1. a mean clinically relevant difference of 5% (2% reduction in the sham CIA group), with a pooled standard deviation (SD) of 8.1%;
2. 2-sided at 5% and 1- at 80%.

Assuming 20% attrition rate, we plan to enrol a total of 216 participants (108 per group).

### 2.7 Randomization and blinding

This trial will use centralized block randomization generated by a computer (using the professional statistical software SAS 9.1.3 to assign a randomized group number to allocate the treatment programme to be completed). The randomization programme and the various parameters established during the process are collectively known as the blind code, which will be sealed with the signature of the person who created the randomization programme and will be maintained by a special research group administrator at Hubei Provincial Hospital of TCM who will not participate in the project. Each eligible patient will receive a group number according to the inclusion sequence and will be assigned to the treatment programme with the corresponding number, which will remain the same throughout the trial. Number jumping or selecting is not allowed. Each number is associated with an emergency envelope with the corresponding number, which will be maintained by a principal researcher in each trial unit.

Blinding will be conducted for all patients, evaluators and statisticians. During the trial, only the persons performing the CIA will be aware of the patient's group assignment. The patients will be assigned in a 1:1 ratio to either the CIA group or the sham control group, which will be called A and B, respectively, in the treatment record report. All information will be locked in the database until the statistical analysis is conducted. This study will utilize an independent treatment room and make appointments with the patients directly to ensure that patients from different groups do not communicate.

### 2.8 Data collection and management

We use Remote Data Capture (RDC) system to perform data entry. The research assistants will fill out all the electrical CRF through RDC system. Researchers will inspect the eCRF, and signed electrically for the eCRF going into effect. The eCRF and the trace of eCRF revising will be left in the Oracle database.

### 2.9 Quality control

This study has a randomized sham-controlled design, to avoid selection bias. The strict control of the inclusion criteria is one of the most important ways to control bias. Formulating specific inclusion criteria and exclusion criteria, strictly restraining the study object within a certain scope and reducing differences contribute to the objectivity of the conclusions. This study will use blinding, i.e., evaluators who are unaware of the characteristics of the groups will evaluate the efficacy and safety; in the summary phase, a third party will perform a blind statistical analysis. Loss of follow-up and adverse effects will be promptly recorded. To ensure a smooth study, specialized clinical training will be provided to all the clinical researchers before the initiation of the trial. Training on the study design and the SOPs will be provided to familiarize each clinical researcher with the studys process and implementation to improve consistency between the internal observations of researchers and the observers to ensure that the conclusions are reliable. Regular team meetings will be held and fully documented. Each centre will design quality control measures and undergo regular inspections to control for centre bias. During the study, the Clinical Research Institute of Hubei Provincial Hospital of TCM will be responsible for quality control, and a specified statistics centre will be responsible for data entry and management.

### 2.10 Data analysis

Categorical variables will be described using frequencies and percentages, continuous variables as means or if the data are skewed, as medians interquartile ranges. Primary outcome and secondary outcomes will be evaluated in the intention-to treat population. Missing data will be imputed by multiple imputation (m = 5). Rate of reduction from baseline or change from baseline as the dependent variables will be analyzed using repeated measured mixed effect model with adjustment for baseline level, sex, age, marriage status, drinking and smoking status, family history of obesity, and duration of disease as fixed effect; center effect will be assessed by including indicator of study sites as random effect for G matrix. Differences between the 2 groups at each visit point will be estimated by including treatment group, visit (categorical variable), an interaction between treatment groups and visit as fixed effect. An overall evaluation will be performed to test linear growth tendency by considering visit as continuous variable. Adjusted mean difference and 95% confidence interval will be presented.

Safety assessments will be conducted in the safety analysis set. Incidence of AEs will be compared using Fisher exact method. The changes in laboratory parameters between baseline and 16 weeks will be analyzed using analysis of covariance. McNemar test will be employed to test inconsistency for the grouping information and blinding assessment will be collected on each participant. To assess the effect the missing data on the primary outcome, we will conduct a sensitivity analysis. The data will be transformed into long format for each participant. In the long format, each row is one time point per participant. So each participant will have data in multiple rows. The rows with missing outcome will be excluded from mixed effect model.

All statistical tests will be conducted using a 2-sided type I error rate of 5%. All analyses will be carried out using the SAS software, version 9.4 TS1M6 (SAS Institute Inc, Cary, NC). Data visualization was conducted by using R version 3.5.1 software (The R Foundation for Statistical Computing, Vienna, Austria (http://www.r-project.org/) with the R packages ggplot2.

## 3 Discussion

For people with simple obesity, acupuncture treatment should be conducted every day or every other day. Because people are often busy with life and work, it is difficult for them to follow the instructions of physicians. CIA changes the pattern of acupuncture treatment in that certain embedded stimulants can stimulate acupoints for about 2 weeks, which significantly increases patient compliance.^[31]^ According to relevant reports, the effect of CIA is similar to that of pricking blood therapy.^[32]^

In our previous studies, it was observed that the body weight, BMI, and fasting blood glucose level decreased after CIA integrated with diet and exercise in the patients with simple obesity.^[33,34]^ However, previous trials were limited by their small size and lack of blinding, and the results were insufficient to determine whether there was a placebo effect; therefore, we designed this study to demonstrate the efficacy and safety of treating simple obesity with CIA. This study follows the guidelines of the STRICTA,^[35]^ the Effectiveness Guidance Document (EGD),^[36]^ and the Consolidated Standards of Reporting Trials (CONSORT).^[37]^ We choose common obesity indexes, including waistline, weight, BMI, hipline, WHR and BFP, scales for assessing psychological status, including the HAD and the SES, scales for assessing the quality of life, including the IWQOL-Lite and the SF-36 as the outcome measurements of efficacy evaluation. For the safety evaluation, general physical examination items (blood pressure, heart rate, routine blood tests, etc.), the incidence of adverse events, and the receptivity to catgut implantation at acupoints will be analyzed.

There are no previous studies of the safety and efficacy of CIA for the treatment of simple obesity using sham control. Referring to previous sham-controlled studies investigating acupuncture for simple obesity and considering the use of blinding and the place of the sham control, the acupoints in the sham control group will be located 1 cun (≈20 mm) from the locations used in the CIA group. The sham acupoints cannot be located at any real acupoints or in the meridians of the body, and no catgut will be embedded during the operation. Due to the characteristics of CIA, we believe that this procedure exhibits adequate blinding. To objectively evaluate the clinical effects of treating simple obesity with CIA, the patients will be unaware of whether they will receive CIA. For both groups, we will also consider ethics. We will provide a basic treatment with diet and exercise to both the treatment group and the control group. If the treatment with CIA has no benefits over sham catgut implantation, the patients will still lose weight and develop good eating and exercise habits.

The instrument for CIA used in this study will be a disposable sterile microinvasive needle, which can improve the quality of catgut implantation and effectively prevent nosocomial infection. According to previous clinical experience, it takes about 2 weeks to completely absorb collagen catgut; therefore, the interval between the 2 treatments will be 2 weeks. The acupoints used in Group A and Group B are alternated based on clinical evidence. To ensure the safety and reliability of this study, all acupuncturists and operators will receive specialized training.

In summary, the primary objective of this study is to demonstrate the safety and efficacy of treating simple obesity with CIA. We expect that this study will provide favourable evidence for the treatment of simple obesity with CIA.

## Acknowledgments

The authors would like to thank all the patients who will participate in this study.

## Author contributions

Zhong-Yu Zhou, Yi-Ran Liu and Xia Chen participated in the conception and design of the trial, and drafted the manuscript. Wei Huang and Dan Wei revised the manuscript. Zhong-Yu Zhou, Dan Wei, Yi-Ting Jin, Yan-Ji Zhang, Yi-Wei Zheng Ying-Rong Zhang, Yi-Ran Liu and Hong-Ling Pan recruited patients and conducted assessments. De-Guang Ding, Yang Jiao and Xia Chen participated in the design of the statistical analysis. Wei Huang and Wei D helped with quality control. All authors read and approved the final revision of the manuscript and approved the final version.

**Conceptualization:** Xia Chen, Yi-Ran Liu, Zhong-Yu Zhou.

**Formal analysis:** De-Guang Ding, Yang Jiao, Yi-Ting Jin, Yan-Ji Zhang, Ying-Rong Zhang, Yi-Ran Liu.

**Project administration:** Dan Wei, Hong-Ling Pan, Yi-Wei Zheng, Zhong-Yu Zhou.

**Supervision:** Wei Huang, Dan Wei, Zhong-Yu Zhou.

**Writing – original draft:** Xia Chen, Yi-Ran Liu, Zhong-Yu Zhou.

**Writing – review & editing:** Wei Huang, Dan Wei.
